# Relation of Dental Infection and Systemic Disease*An extract from an article published in the *Dental Cosmos* for May, at page 485.

**Published:** 1917-06

**Authors:** Edward C. Rosenow


### RELATION OF DENTAL INFECTION AND SYSTEMIC DISEASE.*

BY EDWARD C. ROSENOW, M.D.

I must emphasize the results obtained with the slightly hemolyzing streptococcus isolated from the dead pulp of a tooth and excised muscle from the neck of a patient with myositis and dental neuritis; 71 per cent. of the twenty-four animals injected showed myositis, 50 per cent. dental pulpitis, and 46 per cent. neuritis, chiefly of the dental nerves.

The patient had had repeated attacks for five or six years of neuralgia of the face beginning with severe pain and swelling over the left upper jaw, and spreading to the opposite side. This was associated with soreness in the tooth, especially in the upper jaw, and followed by intense pain and swelling of the glands in the left side

---

* An extract from an article published in the *Dental Cosmos* for May, at page 485.

of the head, neck, and shoulders. Her tonsils were removed four years ago, but the condition was not relieved. One year ago the upper left second molar, showing a blind abscess at the root, was extracted. The left maxillary sinus was drained and a piece of the left turbinate removed, but without relief. The patient had become extremely nervous, at times hysterical, during the paroxysmal pain, and had one or two spells of mental confusion suggesting *petit mal.* The symptoms represent a condition which occurs quite commonly, particularly in neurotic persons.

The upper left first molar appeared to be a "dead" tooth. Owing to the fact that each attack began with swelling of the left upper jaw opposite it, this tooth was extracted. There was found a semilunar eroded area, two mm. in diameter, near the apex of the larger and inner root. The canal of this root was filled with a foul-smelling pus. The erosion was so situated that it was impossible to demonstrate it by roentgenograms. The canals of the other two roots were filled and obliterated and there were no erosions at the apices.

Directly opposite the putrescent pulp was a large cement filling. Smears from the root showed a few Gram-positive diplococci and diphtheroid bacilli, and a few Gram-negative bacilli. The primary cultures in the ascites-dextrose agar and broth gave a pure culture of a slightly hemolyzing streptococcus. After the extraction of the tooth the patient developed an unusually severe attack of neuralgia. Ten days afterward there was freedom from pain. Following this attack there was found definite tenderness of the muscles in the posterior triangle of the left side of the neck, and two distinctly tender nodules just behind the posterior margin of the sternomastoid muscle. One of these tender nodules was thought to be a lymph gland, and a portion of the deeper muscle was excised. The excision of the fascia and muscle precipitated another violent attack of pain and spasm of the muscles of the left side of the neck. The cultures in tall columns of the ascites plain broth of the emulsion of the muscle showed pure culture of short-chained streptococci, and those from the thickened fascia, streptococci and staphylococci. It was thought that these streptococci might be present quite generally on the mucous membrane. Cultures from the nose and pharynx, and of the stool, proved that this was actually the case. Sections of the excised muscles showed marked increase in interstitial tisue, poorly staining nuclei of adjacent muscle fibers, and slight round-cell infiltration. Sections of the fibrous nodule and fascia showed old and young connective tissue, absence of lymphoid tissue, small nests of round cells, plasma cells, and erythrocytes, chiefly around bloodvessels. Stains for bacteria revealed a moderate number of diplococci in or adjacent to the fibrous tissue between the muscle fibers, and a large number of nests of cellular infiltration within and surrounding a smallsized bloodvessel in the center of the fibrous nodule.

This is, as far as can be determined, the first experimental demonstration of the production of dental neuritis and pulpitis by intravenous injection of bacteria, and is in accord with the view held by some observers that infection of the pulps of sound teeth is always hematogenous in origin. Its occurrence warrants search for a focus in the tonsils or elsewhere. In the light of these experimental results, is it not likely that abscesses about devitalized teeth frequently have the same origin? The results in this case show also that a foul pulp with no abscess at the apex and having negative roentgen findings, or with only a small granuloma, is sufficient to act as a focus of infection. I mention this point particularly because of the erroneous impression in the minds of many, that negative roentgen findings of the jaws exclude the presence of foci of infection in this region.

In one case of acute pyorrhea the bacteria in the first culture injected intravenously into a rabbit produced hemorrhage and edema of the peridental membrane, suggesting that Riggs' disease may not always be the result of the local condition of the teeth or gum, but may in some instances be due to hematogenous infection by bacteria having this peculiar localizing power. However, further experiments are necessary before conclusions can be drawn.

In another case similar studies indicated the etiologic relationship between a severe pyorrhea and heart-block.

### NECESSITY OF CLOSER AFFILIATION BETWEEN THE MEDICAL AND DENTAL PROFESSIONS

The relationship between dental infections and various systemic diseases is demonstrated. The eradication of these foci of infection, often symptomless, is indicated. The exact methods to be followed in ridding patients of existing dental foci of infection must be decided in each individual case. A severe pyorrhea, if incurable by other means, calls for extraction of the affected teeth. Pulpless and abscessed teeth are to be regarded as a serious menace to continued health, and, with relatively few exceptions, should be extracted. A devitalized tooth, even if properly filled, because of a diminished blood supply is necessarily a point of lowered resistance to infection. I do not presume to pass judgment as to whether root-canals can or can not be filled properly. In some instances it is mechanically impossible. To devitalize teeth is therefore dangerous, even in the hands of the most skilled.

As pointed out above, the occurrence of pulpitis in a sound tooth or in one whose root-canal is properly filled, speaks for the existence of a focus of infection in some other part of the body. Not too much should be expected from the removal of a focus, especially in chronic conditions, because a similar condition may be present in inaccessible foci and in others too small to be detected. Moreover, recovery may be made difficult by local tissue sensitivity or peculiar mechanical conditions, and living bacteria in a metastatic lesion may continue the process independently of the focal source. A focus is of importance not only as affording an entranceway for bacteria, but also as a place where varying affinities may be acquired. Bacteria are exceedingly small, and may enter the blood without visible signs on the surface. We must not over-emphasize the mechanical factors and neglect the point of greatest importance—namely, the infecting power of the bacteria at hand.

The results detailed call for a closer affiliation of the dental and medical professions. The dentist of the future should not be isolated in a small office over a drug store, his chief work being to devitalize and fill teeth without regard to the general health. Properly trained dentists should be a part of the public school system and hospitals, in which instruction in the care of teeth should be given. The dentist should be closely associated with a group of physicians so that patients may be given the benefit of a coordinate group of specialists with a minimum loss of time and expense. Moreover, it seems to me, these results call for a public health propaganda so that the people may be fully informed of the dangers to their continued health that arise from infected teeth, and how to avoid them. Prophylaxis should be the watchword.

